# Abnormally Small Neuromuscular Junctions in the Extraocular Muscles From Subjects With Idiopathic Nystagmus and Nystagmus Associated With Albinism

**DOI:** 10.1167/iovs.16-19129

**Published:** 2016-04-19

**Authors:** Linda K. McLoon, Christy L. Willoughby, Jill S. Anderson, Erick D. Bothun, David Stager, Joost Felius, Helena Lee, Irene Gottlob

**Affiliations:** 1 Department of Ophthalmology and Visual Neurosciences University of Minnesota, Minneapolis, Minnesota, United States; 2 Department of Neuroscience, University of Minnesota, Minneapolis, Minnesota, United States; 3 Department of Pediatrics, Minneapolis, Minnesota, United States; 4 Pediatric Ophthalmology and Adult Strabismus, Plano, Texas, United States; 5 Retina Foundation of the Southwest, Dallas, Texas, United States; 6 Clinical and Experimental Sciences, Faculty of Medicine, University of Southampton, Southampton, United Kingdom; 7 The University of Leicester Ulverscroft Eye Unit, Department of Neuroscience, Psychology and Behaviour, Leicester, United Kingdom

**Keywords:** nystagmus, neuromuscular junction, albinism, acetylcholine receptor, extraocular muscles

## Abstract

**Purpose:**

Infantile nystagmus syndrome (INS) is often associated with abnormalities of axonal outgrowth and connectivity. To determine if this manifests in extraocular muscle innervation, specimens from children with idiopathic INS or INS and albinism were examined and compared to normal age-matched control extraocular muscles.

**Methods:**

Extraocular muscles removed during normal surgery on children with idiopathic INS or INS and albinism were immunostained for neuromuscular junctions, myofiber type, the immature form of the acetylcholine receptor, and brain-derived neurotrophic factor (BDNF) and compared to age-matched controls.

**Results:**

Muscles from both the idiopathic INS and INS and albinism groups had neuromuscular junctions that were 35% to 71% smaller based on myofiber area and myofiber perimeter than found in age-matched controls, and this was seen on both fast and slow myosin heavy chain isoform–expressing myofibers (all *P* < 0.015). Muscles from subjects with INS and albinism showed a 7-fold increase in neuromuscular junction numbers on fast myofibers expressing the immature gamma subunit of the acetylcholine receptor. The extraocular muscles from both INS subgroups showed a significant increase in the number and size of slow myofibers compared to age-matched controls. Brain-derived neurotrophic factor was expressed in control muscle but was virtually absent in the INS muscles.

**Conclusions:**

These studies suggest that, relative to the final common pathway, INS is not the same between different patient etiologies. It should be possible to modulate these final common pathway abnormalities, via exogenous application of appropriate drugs, with the hope that this type of treatment may reduce the involuntary oscillatory movements in these children.

Infantile nystagmus syndrome (INS) is an eye movement disorder characterized by conjugate, involuntary oscillations of the eyes that can result in substantially decreased visual acuity for most patients. Despite a prevalence of 0.03% to 0.24% in the population,^1,2^ there currently is no effective cure for this visually debilitating disorder.^3^ Often diagnosed in the first 2 to 3 months of life, a large proportion of INS patients have no known contributing factor for their gaze holding disorder (idiopathic INS; 2.9 per 10,000 people under age 18).^2^ However, INS is commonly associated with afferent visual defects including bilateral optic nerve hypoplasia, aniridia, achiasma, retinal dystrophies, and albinism.^4^ Albinism in particular has a strong association with INS, with approximately 90% of individuals with albinism also having nystagmus (88%^5^; 89.9%^6^; 89%^7^).^5–7^ Even patients with albinism and no clinically detectable nystagmus were shown to have significant saccadic instabilities.^8^ While there are a number of genes associated with different forms of albinism, the lack of pigmentation in these individuals leads to foveal hypoplasia and abnormal routing of the optic nerves.^9,10^ In addition, it has been shown in animal models of albinism that the optic nerves contain a population of abnormal axons.^11^ Thus, lack of melanin has been described as a primary cause of the resultant nystagmus and reduced visual acuity common to albinism.^12^ There is other evidence that abnormalities in axonal outgrowth during development play a critical role in development of idiopathic INS. Recent work has shown that approximately 25% of children with idiopathic INS have a mutation in the *FRMD7* gene,^13^ which functions in controlling nerve outgrowth.^14^ The linkage between albinism and nystagmus is further strengthened by the demonstration that albino mice and rabbits also have nystagmus-like eye movements.^15,16^

There are currently very few treatments for reducing the involuntary oscillatory movements associated with INS.^17^ Most drug treatments involve systemic delivery of gamma aminobutyric acid (GABA) agonists and potassium channel blockers, as well as a variety of other drugs; these treatments often have unwanted systemic side effects.^18^ The two main surgical procedures performed on the extraocular muscles for INS may improve head posture and alter the nystagmus waveform but do not eliminate the abnormal eye oscillations. The primary goal of the Kestenbaum-Anderson surgical procedure^19,20^ is to improve head posture by moving the null point, defined as the eye position where the nystagmus intensity is the least and vision is the best, into primary gaze. This surgery involves large resections of selected extraocular muscles and recessions of their antagonists. The muscles produced as surgical waste from this procedure were the primary source of material examined in the present study. Tenotomy surgery also does not eliminate oscillatory movements.^21^ These approaches focus on the symptoms, often the anomalous head posture, rather than a potential “cure.” Development of more effective treatments for nystagmus is significantly hampered by our lack of understanding of the primary cause of the involuntary oscillatory movements in a number of types of childhood-onset nystagmus.

In a previous study, we demonstrated that the extraocular muscles (EOMs) from idiopathic INS patients contained centrally nucleated myofibers, a sign of denervation/reinnervation, as well as decreased density of innervating nerve fibers and neuromuscular junctions (NMJ).^22^ Our goal in the present study was to determine if similar findings would be seen in the EOMs from patients with albinism and INS. Our original study of EOM surgical waste specimens obtained from subjects with idiopathic INS at the time of surgery suggested changes that could reflect either retention of immature NMJ properties or a process of escalated remodeling of the NMJ. To address this second question, the overall NMJ length and area on both fast and slow myofibers were determined. The expression patterns of the immature gamma subunit on myofibers were assessed morphometrically in both subjects with idiopathic INS and subjects with INS associated with albinism, and these data were compared to those derived from age-matched control EOM samples. In addition, EOMs from all three groups of subjects were examined for expression of brain-derived neurotrophic factor (BDNF).

## Methods

The EOM specimens from patients with INS, either idiopathic or with associated albinism, were obtained as surgical waste at the time of their regularly scheduled surgery, either at the University of Minnesota Hospital or at the University Hospitals of Leicester, National Health Service Trust. All research was approved by the Institutional Review Board of each university and complied with the tenets of the Declaration of Helsinki for the use of human tissue in research. A diagnosis of albinism was based on the presence of iris transillumination defects, crossed asymmetry on visual evoked potential (VEP) recordings, and typical foveal hypoplasia on optical coherence tomography (OCT) imaging. For idiopathic INS, the specimens had typical eye movement recordings, normal OCTs, and normal VEP and electroretinogram (ERG) examinations. All specimens were given a unique number so that they were de-identified, were embedded in tragacanth gum, and were frozen in methylbutane chilled to a slurry on liquid nitrogen. Tissue was stored at −80°C until sectioned. Patient demographics and clinical characteristics are listed in the Table. Control specimens were obtained from a variety of sources and have been described in detail previously.^23^

**Table:**
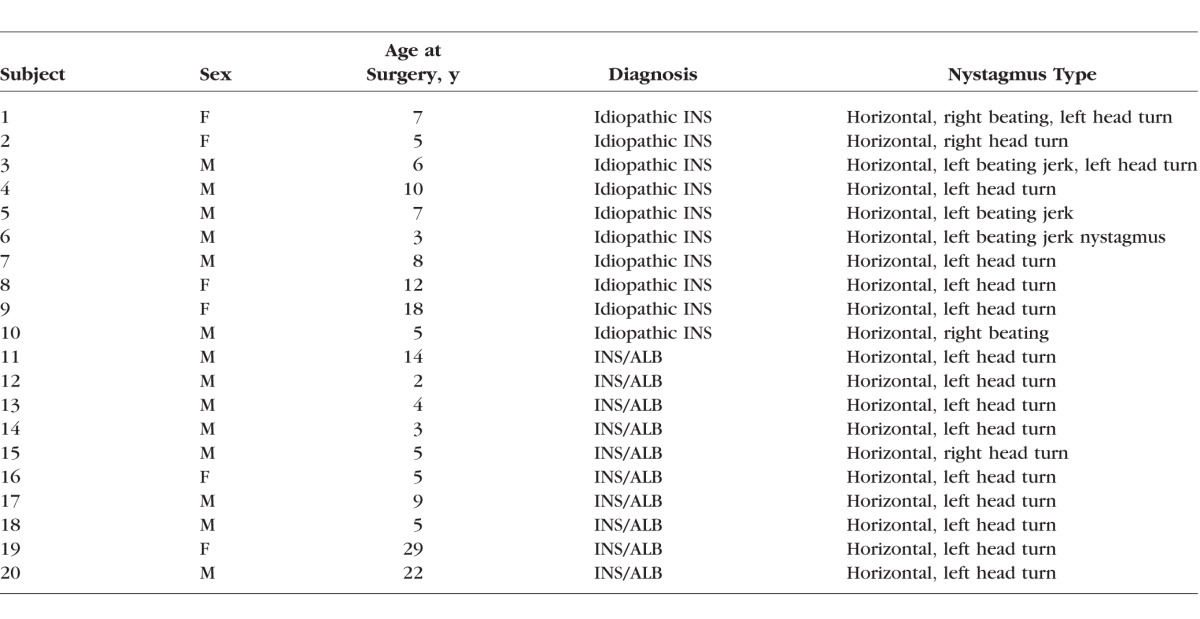
Characteristics of the Subjects With Idiopathic INS and INS Associated With Albinism (INS/ALB)

All muscle samples were sectioned frozen at 10 μm. Sections were stained for hematoxylin and eosin and used for determination of mean myofiber cross-sectional area and percent of myofibers with central nucleation. Tissue sections were processed for the immunohistochemical visualization of the slow myosin heavy chain isoform (MyHC) (1:1000; Hybridoma Bank; University of Iowa, Iowa City, IA, USA), neurofilament protein (smi-31; 1:30,000; BioLegend, Dedham, MA, USA), and the gamma subunit of the acetylcholine receptor (γAChR) (1:300; Abcam; Cambridge, MA, USA) using our standard protocol, summarized here. Sections were rinsed in 0.01 M phosphate-buffered saline (PBS) with 0.1% Triton X-100 (antibody buffer), pH 7.4, blocked for 1 hour in the appropriate serum at 10% in PBS, and incubated in primary antibody diluted in antibody buffer overnight at 4°C in humid chambers. The sections were rinsed in PBS, followed by incubation in a secondary antibody conjugated with either Cy3 (1:500; Jackson ImmunoResearch Laboratories; West Grove, PA, USA) or Dylight 405 (1:100; Jackson ImmunoResearch Laboratories) and diluted in antibody buffer. In addition, selected sections were washed, followed by incubation with α-bungarotoxin conjugated to Alexa Fluor 488 overnight. Sections were washed and mounted with Vectashield (Vector Laboratories, Burlingame, CA, USA).

Morphometric analyses were performed using either Bioquant (Nashville, TN, USA) or ImageJ software (http://imagej.nih.gov/ij/; provided in the public domain by the National Institutes of Health, Bethesda, MD, USA). Myofiber mean cross-sectional area and rate of central nucleation were determined by manual tracing/counting on at least three sections from different regions of the muscle specimen on a minimum of 200 myofibers per slide. These were averaged for each specimen and then averaged for all specimens in each of the three groups. Neuromuscular junction density and length, length relative to myofiber perimeter, and NMJ area compared to myofiber area were determined on four or five specimens in EOM from controls, subjects with idiopathic INS, and subjects with INS and albinism. Perimeter was determined by manually circling individual myofibers using length as the metric. Every NMJ was characterized for each complete cross section, and at least three slides were imaged and analyzed per muscle specimen.

Within-subject comparisons were made between paired muscles from the subjects with idiopathic INS and those with INS and albinism; no significant difference was seen between them, so data from a single subject were averaged for all morphometric analyses.

In addition, the three groups of specimens were examined for expression of BDNF using immunochemistry, with a sheep anti-BDNF primary antibody (1:50, Abcam) and a donkey anti-sheep Cy3 secondary antibody (1:200; Jackson ImmunoResearch Laboratories).

Data are reported as mean ± standard error of the mean. Statistical analyses were performed using ANOVA with a Tukey's post hoc multiple comparison test for multiple group comparisons. Data were considered to be significantly different if *P* < 0.05.

## Results

**Figure 1:**
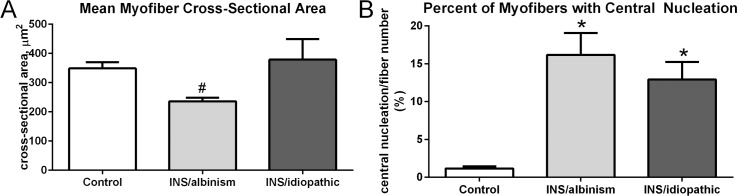
(**A**) Mean cross-sectional areas of EOM myofibers from control muscles compared to muscles from subjects with INS and albinism and compared to subjects with idiopathic INS. #Significant difference between INS and albinism compared to INS only. (**B**) Percent of myofibers with central nucleation in the EOM from control muscles compared to EOM from subjects with INS and albinism and compared to subjects with INS and no known sensory afferent defects. *Significant difference from control. *n* = 10 for all groups.

The EOM specimens from subjects with INS and albinism had decreased myofiber cross-sectional areas, averaging 235.4 ± 12.7 μm^2^ when all myofibers were included, compared to the specimens from control subjects and those with idiopathic INS, averaging 348.5 ± 21.22 and 378.4 ± 70.72, respectively (Fig. 1A). In addition, in both groups there was a significant increase in numbers of myofibers with central nucleation, 12.9 ± 2.3% for specimens from subjects with idiopathic INS and 16.18 ± 2.88% for those with INS associated with albinism, compared to normal age-matched control EOM, where only 1.28 ± 0.27% of myofibers contained centralized nuclei (Fig. 1B).

**Figure 2:**
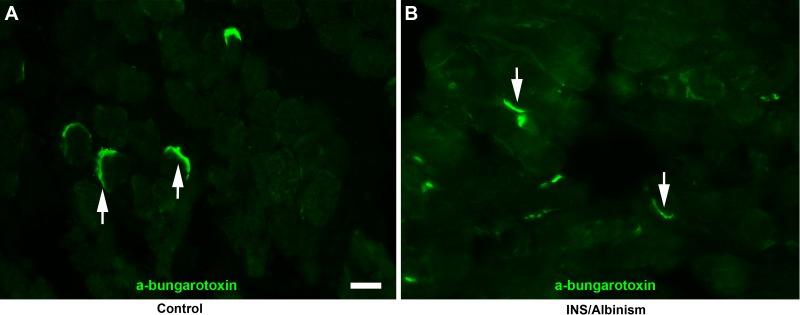
Neuromuscular junctions visualized with α-bungarotoxin conjugated to Alexa Fluor 488 in (**A**) an age-matched control EOM and (**B**) an EOM from a subject with INS and albinism. *Arrows* indicate neuromuscular junctions. *Scale bar*: 50 μm.

**Figure 3:**
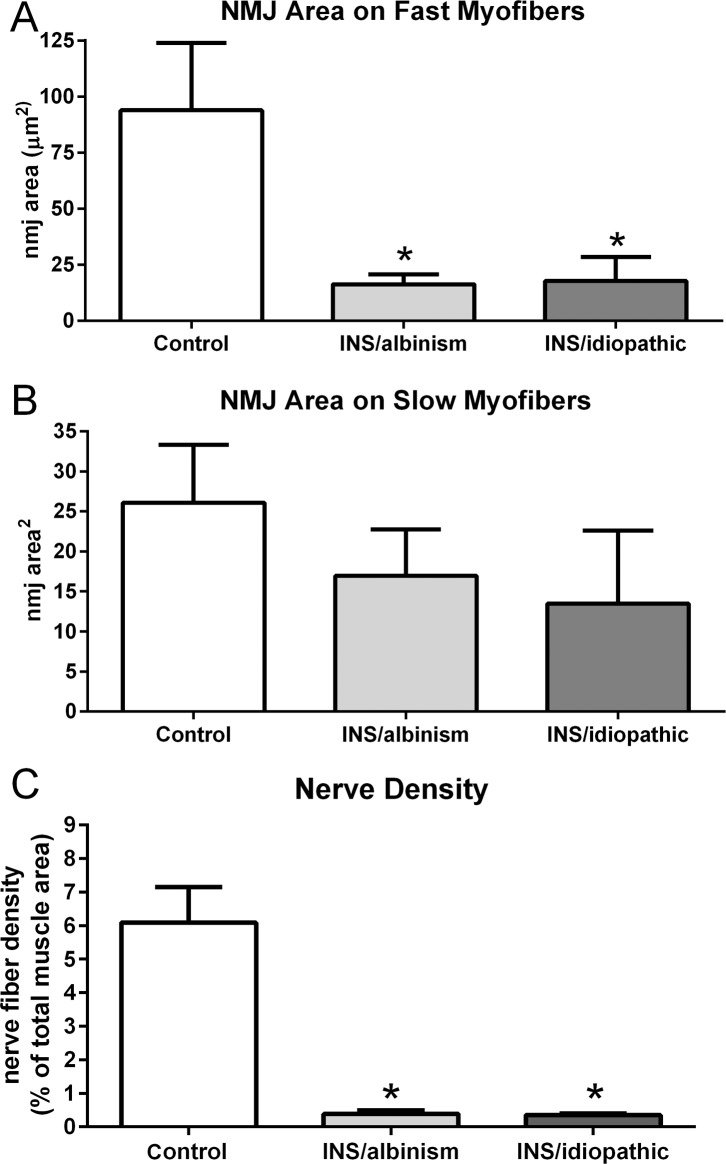
Mean neuromuscular junction (NMJ) areas on (**A**) fast myofibers, (**B**) slow myofibers, and (**C**) nerve density comparing subjects with INS and albinism, subjects with idiopathic INS, and age-matched controls. *Significant difference from control. *n* = 4 for controls, *n* = 5 for idiopathic INS and INS and albinism specimens.

When the EOM from subjects with idiopathic INS and INS associated with albinism were examined, it appeared that the NMJs were smaller than in the age-matched control specimens (Fig. 2). Mean NMJ areas were calculated for EOM from both sets of subjects as well as control muscles for myofibers expressing either fast or slow MyHC (Figs. 3A, 3B). In the specimens from individuals with idiopathic INS or INS associated with albinism, the mean areas of the NMJs found on fast MyHC–positive myofibers were 68.7% and 73.4% smaller, respectively, significantly different from the age-matched control values (Fig. 3A; both at *P* = 0.0001). There were no significant differences between the mean NMJ areas on slow myofibers in any of the three groups of specimens (Fig. 3B). The decreased areas of NMJs correlated with the measurements of nerve density (Fig. 3C). As shown previously for specimens from subjects with idiopathic INS,^22^ the EOM from subjects with INS associated with albinism also had significantly reduced nerve fiber density compared to control values, at 0.39 ± 0.1% compared to control values of 6.09 ± 1.06% (Fig. 3C).

**Figure 4:**
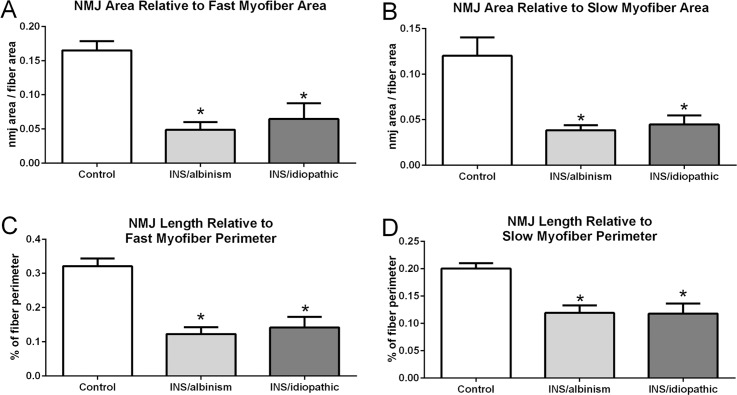
(**A**) Mean neuromuscular junction (NMJ) area relative to fast myofiber area. (**B**) Mean NMJ area relative to slow myofiber area. (**C**) Mean NMJ length relative to fast myofiber perimeter. (**D**) Mean NMJ length relative to slow myofiber perimeter. All comparisons are between subjects with INS and albinism, subjects with idiopathic INS, and age-matched controls, *Significant difference from age-matched controls. *n* = 4 for controls, *n* = 5 for idiopathic INS and INS and albinism specimens.

However, myofibers expressing slow and fast MyHC isoforms are not uniform in cross-sectional area. To determine if there was a difference relative to the size of slow compared to fast myofibers, these values were recalculated based on NMJ area relative to myofiber area and NMJ length relative to myofiber perimeter (Fig. 4). This analysis showed that relative to fiber size, whether perimeter or area, for both fast and slow myofibers the NMJs were significantly smaller on the muscles from the subjects with idiopathic INS and INS associated with albinism than age-matched controls (Fig. 4). These differences in NMJ size compared to age-matched control muscles were, on average, between 62.5% and 71.3% smaller for fast myofibers based on fiber area (*P* = 0.007 for idiopathic INS and *P* = 0.0012 for INS associated with albinism) and 60% smaller based on perimeter (*P* = 0.003 for idiopathic INS and *P* = 0.0006 for INS associated with albinism) (Figs. 4A, 4C). For the slow myofibers, the NMJs were, on average, 67% smaller based on myofiber area (*P* = 0.0039 for idiopathic INS and *P* = 0.0009 for INS associated with albinism), and 35% to 37% smaller for slow myofibers based on fiber perimeter (*P* = 0.015 for idiopathic INS and *P* = 0.009 for INS associated with albinism) (Figs. 4B, 4D).

**Figure 5:**
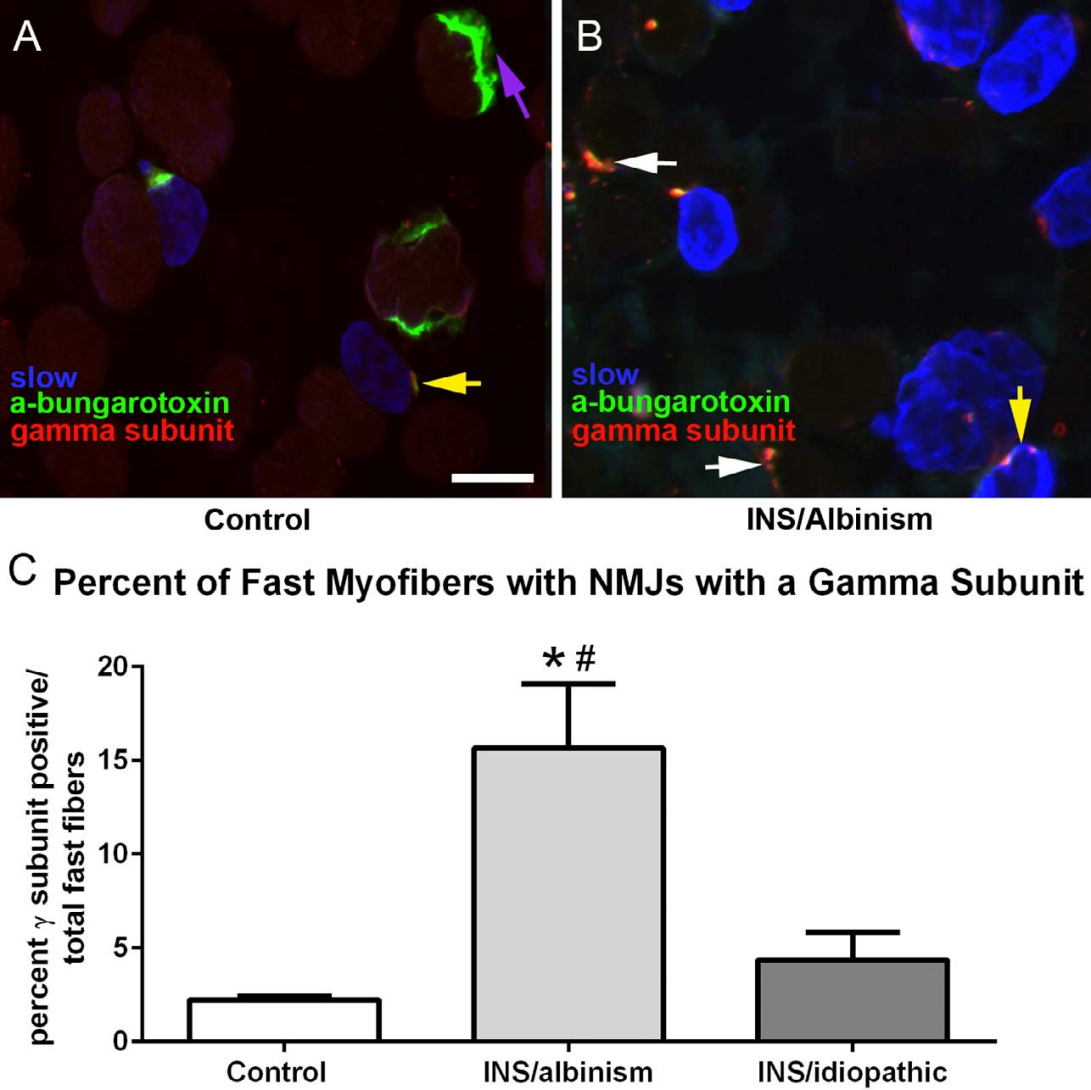
(**A**) Photomicrograph of a control EOM immunostained with α-bungarotoxin bound to Alexa Fluor 488 (*green*), the gamma subunit of the nicotinic acetylcholine receptor (*red*), and slow myosin heavy chain isoform (*blue*). (**B**) Photomicrograph of EOM from a subject with INS and albinism immunostained as in (**A**). *White arrows* indicate neuromuscular junctions positive for both the gamma subunit and α-bungarotoxin on fast myofibers. *Yellow arrows* indicate neuromuscular junctions that are positive for both the gamma subunit and α-bungarotoxin on slow fibers. The *purple arrow* indicates a neuromuscular junction stained with α-bungarotoxin but negative for the gamma subunit. *Bar* is 20 μm. (**C**) Morphometric analysis of the percentage of fast myofibers that had neuromuscular junctions positive for the immature gamma subunit of the nicotinic acetylcholine receptor. *Significant difference from control. #Significant difference from idiopathic INS.

The small NMJs suggested that they might not be fully mature or might be undergoing remodeling. To test this hypothesis, the NMJs were examined for expression of the immature γAChR, which is normally not expressed in the NMJs of mature skeletal muscle. It should be noted that in normal mature EOM, the NMJs on the majority of the slow myofibers express the γAChR on the en grappe endings that are commonly found on this fiber type.^24,25^ As expected, we found many en grappe endings positive for the gamma subunit on slow-positive myofibers as well as en plaque endings on fast-positive myofibers that stained for α-bungarotoxin but appeared to be negative for the gamma subunit (Fig. 5A). In the EOM from subjects with INS associated with albinism, fast myofibers with NMJs positive for the immature gamma subunit were quite noticeable (Fig. 5B). Morphometric analysis of EOM from control, idiopathic INS, and INS associated with albinism muscle specimens demonstrated a significant 7-fold increase in the percent of fast myofibers with gamma-positive NMJs in the EOM from subjects with INS associated with albinism (Fig. 5C), from 2.23 ± 0.2% to 15.67 ± 3.4% compared to control subjects. While there was a 2-fold increase, to 4.37 ± 1.48%, in the percentage of fast fibers with gamma-positive NMJs in the EOM of subjects with idiopathic INS, this difference was not significantly different from control. The percentage of fast fibers with gamma-positive NMJs was also significantly different between the EOM of subjects with INS associated with albinism and idiopathic INS (Fig. 5C).

**Figure 6:**
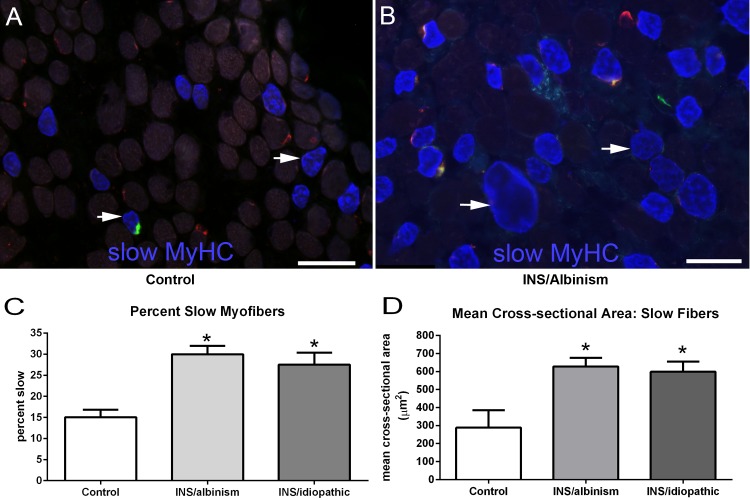
(**A**) Photomicrograph of EOM from a control subject and (**B**) a subject with INS and albinism immunostained with α-bungarotoxin bound to Alexa Fluor 488 (*green*), the gamma subunit of the nicotinic acetylcholine receptor (*red*), and slow myosin heavy chain isoform (MyHC) (*blue*). *Bar* is 50 μm. (**C**) Morphometric analyses of the percent of myofibers positive for the slow MyHC in the EOM from all three cohorts. (**D**) Morphometric analysis of the mean cross-sectional area of slow MyHC–positive myofibers in the EOM from all three cohorts. *Significant difference from control. *n* = 9 for all subject cohorts.

In the process of examining NMJs, it appeared that there was a change in the proportions of fast and slow myofibers in the EOM from subjects with INS compared to controls (Figs. 6A, 6B). Quantification showed that there was a significantly higher percentage of slow myofibers in the EOM from the subjects with INS associated with albinism compared to control, at 29.98 ± 1.97% compared to the control level of 15.04 ± 1.8% (Fig. 6C). The EOM from subjects with idiopathic nystagmus contained 27.53 ± 2.82% slow-positive myofibers, which also was significantly different from controls. When mean cross-sectional areas were compared, the slow myofibers in specimens from subjects with INS associated with albinism were significantly larger than control values, at 627.9 ± 48.14 μm^2^ compared to 288.5 ± 97.13 μm^2^ in control specimens (Fig. 6D). The mean cross-sectional areas for the muscles from those with idiopathic INS were 599.1 ± 56.2 μm^2^. This was also significantly different from control specimens.

**Figure 7:**
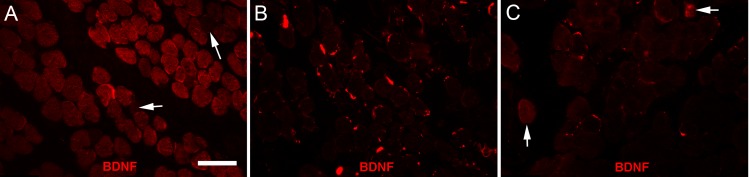
(**A**) Normal EOM immunostained for the expression of brain-derived neurotrophic factor (BDNF). Note that the majority of myofibers were positive for BDNF, although negative myofibers were seen (*arrows*). (**B**) Representative section of an EOM from an INS/albinism subject immunostained for BDNF. Note that the fibers shown are all negative in this field. (**C**) Representative section of an EOM from an idiopathic INS subject immunostained for BDNF. Note that the fibers shown are all negative except the two indicated by the *arrows*. *n* = 9 for all subject cohorts. *Scale bar*: 50 μm.

All three cohorts of muscles were examined for expression of BDNF (Fig. 7). Myofibers were positive for BDNF in all the control muscles, with a relatively rare negative myofiber interspersed between the largely positive myofiber population (Fig. 7A). In contrast, the EOMs examined from the subjects with idiopathic INS and INS and albinism were largely negative for BDNF expression (Figs. 7B, 7C). In some of the specimens from subjects with idiopathic INS, small numbers of BDNF-positive myofibers could be seen (Fig. 7C, arrow), but these were extremely rare in the specimens from the subjects with INS and albinism.

## Discussion

The EOM from subjects with INS and albinism and idiopathic INS were significantly different from the control specimens in a number of ways. In the subjects with INS and albinism, these differences included smaller NMJ compared to control EOM, greater numbers of NMJ on fast myofibers expressing the immature γAChR, a higher percentage of and larger slow myofibers in these muscles, and the absence of BDNF-positive myofibers. Similar differences were seen in the specimens from subjects with idiopathic INS, except that there was only rare expression of the immature acetylcholine receptor on fast myofibers.

These results make sense in light of the broader context of conditions with abnormal or aberrant nerve growth that often coexist in children with idiopathic INS. The only gene thus far associated with idiopathic INS is *FRMD7*,^13^ and a mutation in this gene results in altered neurite outgrowth and development.^14,26^ Similarly, albinism is associated with distinct alterations to optic axon pathfinding from the retina to the brain^27^ but there are also abnormal connections within other systems including the auditory system.^28^ Another condition associated with nystagmus is optic nerve hypoplasia, and a number of studies have demonstrated that it is associated with higher visual pathway abnormalities as well as developmental abnormalities of midline brain structures and generalized white matter hypoplasia.^29,30^ Infantile nystagmus syndrome is associated with achiasma,^31^ which similarly to albinism represents a miswiring in the pattern of optic axon projections to the brain. The unresolved question is whether there are concomitant axon growth alterations in the motor output that controls eye movements.

The oculomotor innervational status in subjects with nystagmus associated with albinism has not been characterized. Two case reports demonstrated an association between Duane's syndrome, characterized by lack of innervation to the lateral rectus muscle by the abducens nerve, and albinism.^32.33^ In our earlier study we showed that in the EOM from subjects with idiopathic INS there was a significant reduction in the density of NMJs that correlated with decreased density of innervating nerve fibers.^22^ These studies suggest that there may be a common mechanism in these patient populations that affects the normal patterning of innervation.

In addition to an overall reduction in the density of innervating motor axons, a significant decrease in the size of NMJ and an increase in the number of NMJ on fast myofibers that expressed the immature γAChRs were seen. While the functional sequelae of these changes in innervational status in these children were involuntary oscillatory movements of the eyes, the mechanism causing these innervational changes in altering eye stability is unclear. These innervational differences may be a primary cause of the oscillatory movements, or may be a secondary adaptation to the nystagmus. Nonetheless, these differences would likely alter eye muscle function. The literature on the role of neuromuscular maturation on muscle function suggests that immature motor systems controlling limb muscles are characterized by unstable temporal parameters and dissociated firing patterns.^34^ In other studies where experimental manipulations resulted in smaller NMJ, muscles were weaker after either nerve or direct muscle stimulation when compared to normal controls.^35^ Still other studies showed decreased frequency of neurotransmitter release,^36^ as well as both reduced synaptic efficiency and reduced force generation.^37,38^ These could all play a role in producing involuntary oscillatory eye movements.

The increase in the size and proportion of slow myofibers in the EOM from the subjects with both INS and albinism and idiopathic INS is very interesting. Fast and slow myofibers have distinctly different contractile profiles, as slow myofibers contract more slowly but produce more sustained force before fatiguing.^39^ It is impossible to ascertain from single EOM specimens whether this alteration in slow myosin–positive myofibers is a primary or compensatory event in these children with infantile nystagmus and albinism. If it is secondary, this would suggest an attempt of the ocular motor system to drive the EOM in these subjects toward a phenotype that could have the potential to dampen the involuntary oscillatory eye movements. Compensatory changes in myosin isoform expression are common after various types of perturbations of the EOM. For example, significant alterations in fiber type densities in the EOM were seen in animal models after various types of treatments, including strabismus surgery^40,41^ or injection of botulinum toxin A.^42^ Studies have shown a differential effect on fast compared to slow myofibers after sustained treatment with bone morphogenetic protein-4 (BMP-4)^43^ and an effect only on slow myofibers after sustained treatment with BDNF.^44^ We are currently assessing if changes in slow myofiber number and size are also found in albino animal models, and if so, we should be able to test if modulation of these differences alters the pattern of nystagmus found in these animals.

Interestingly, when endogenous expression of BDNF was examined in control human muscles, it was preferentially increased in the proximal and distal regions of the EOM and essentially absent in the midbelly region of the muscle fibers.^44^ Differential expression of IGF-1 has also been demonstrated in the EOM, but in a completely different pattern,^45^ suggesting a tight regulation of neurotrophic expression patterning in the maintenance of normal EOM structure and function. The proximal and distal regions of the EOM contain mainly en grappe NMJ that are more involved in the tonic or step phase of muscle contraction.^46^ This geographic linkage in BDNF localization suggests that BDNF could play a role in the formation and/or maintenance of the en grappe synapses. The relative absence of BDNF expression in the EOMs of both INS groups compared to that seen in the control EOM is interesting in light of the roles BDNF can play in neuromuscular structure and function. Alterations of BDNF may be a precipitating factor in the subjects with INS and albinism and in idiopathic INS. This is supported by the demonstration that after experimental abducens nerve section and application of only BDNF, only the tonic firing component of abducens motor neuron behavior returned.^47^ Thus, in its absence an increase in fast motor neuron firing with decreased tonic firing would be predicted. Further work is needed to clarify the role that the pattern of BDNF expression and localization plays in EOM structure and function in the EOM of both subjects with idiopathic INS and those with INS and albinism.

In summary, we found that the EOM specimens from subjects with INS and albinism differed from normal age-matched controls in five distinct ways: decreased innervational density, increased density of slow myofibers, increased size of slow myofibers, decreased size of NMJs, and increased numbers of fast fibers whose NMJs expressed the immature γAChR (Fig. 7). While EOM specimens from subjects with idiopathic INS also showed decreased innervation and decreased size of NMJs, the significant increase in immature synapses on fast myofibers was unique to those specimens from the subjects with INS associated with albinism. Specimens from subjects with INS associated with optic nerve hypoplasia also did not show increases in immature synapses on fast myofibers (McLoon LK, et al. *IOVS* 2015;56:ARVO E-Abstract 3998). These studies suggest that, relative to the final common pathway, INS is not the same between different patient groups. It should be possible to modulate these final common pathway abnormalities via exogenous application of neurotrophic factors. Strategies might include methods that alter the muscle contraction profile by modification of myosin heavy isoform expression patterns such as occurs with BMP-4^43^ or BDNF,^44^ reducing insulin-like growth factor levels with antibodies or binding proteins,^48^ or various pharmacologic agents such as resveratrol, which have been shown to produce a fast to slow myofiber type shift.^49^ If shown to be effective in animal models of INS, one of these agents may serve as a potential approach to reduce the involuntary oscillatory movements in these children.
